# Acute Liver Injury with Severe Coagulopathy in Marasmus Caused by a Somatic Delusional Disorder

**DOI:** 10.1155/2011/176894

**Published:** 2011-09-06

**Authors:** Lance L. Stein, Arun B. Jesudian

**Affiliations:** ^1^Piedmont Transplant Institute, Piedmont Hospital, 1968 Peachtree Road NW, 77 Building, 6th Floor, Atlanta, GA 30309, USA; ^2^Division of Gastroenterology, New York Presbyterian Hospital, Weill Medical College of Cornell University, 1305 York Avenue, 4th Floor, New York, NY 10021, USA

## Abstract

Marasmus is a severe form of protein-calorie malnutrition characterized by the depletion of fat stores, muscle wasting, and the lack of edema. In developed countries, marasmus is often the result of anorexia nervosa. Abnormal transaminases with liver synthetic dysfunction have rarely been reported with anorexia nervosa. To our knowledge, we report the first detailed case of acute liver injury with severe coagulopathy (INR > 1.5) in a patient with marasmus due to self-induced calorie restriction caused by a somatic delusional disorder. This case highlights the severity of liver injury that may occur with significant weight loss from self-induced calorie restriction and the rapid normalization of this injury with treatment. It is important for clinicians to be aware of patterns of acute liver injury in patients with severe protein-calorie malnutrition, regardless of the underlying cause.

## 1. Introduction

Marasmus is a severe form of protein-calorie malnutrition characterized by the depletion of subcutaneous fat stores, muscle wasting, and the absence of edema. In developed countries, marasmus is commonly the result of anorexia nervosa. Complications of marasmus include bradycardia, hypotension and hypothermia. Abnormal liver transaminases with liver synthetic dysfunction have rarely been reported to occur in anorexia nervosa. To our knowledge, we report the first case of acute liver injury with severe coagulopathy (INR > 1.5) in a patient with marasmus caused by a somatic delusional disorder.

## 2. Case Presentation

**Figure 1:**
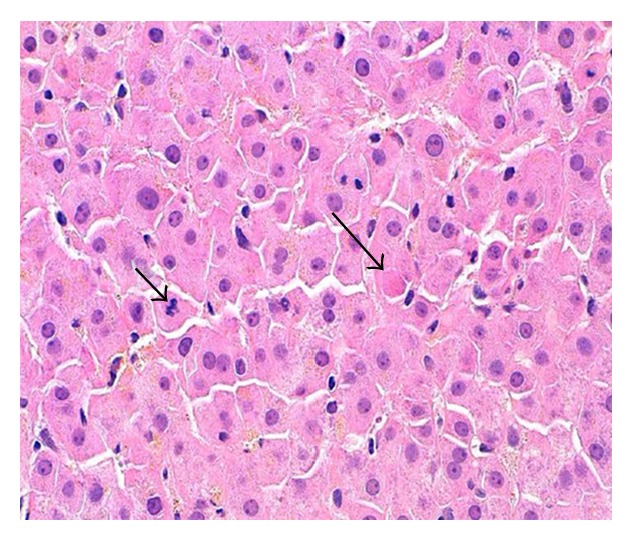
H&E stain at 400x shows mild acute hepatitis with rare acidophil bodies (long arrow) and increased mitotic figures (short arrow) in the hepatic lobule.

A 43-year-old woman was admitted for the evaluation of a 25 kg weight loss resulting in severe protein malnutrition due to intentional calorie restriction (300 Kcal/day) for six months. She had no previous medical or surgical history and was not receiving medications. The patient believed that she developed an “autoimmune disease” that improved with self-induced calorie restriction. On presentation, the patient weighed 30.1 kg (BMI 11), temperature 98.6°F, heart rate 75 bpm, and blood pressure 105/68 mmHg. She was markedly cachectic with temporal wasting, scaphoid abdomen, and normal liver span without stigmata of cirrhosis. She was normally oriented and asterixis was absent. Her admission laboratory testing revealed AST (2895 U/L), ALT (1868 U/L), alkaline phosphatase (460 U/L), bilirubin (2.9 mg/dL), albumin (3.7 g/dL), INR (1.9), amylase 188, lipase 322, CPK 535, normal TSH, negative viral hepatitis and autoimmune serologies, ceruloplasmin (17 mg/dL), and elevated 24-hour urine copper (117 mcg). Abdominal ultrasound revealed a heterogeneous liver with patent vessels. A slit lamp exam failed to reveal Kayser-Fleischer rings. Psychiatry consultation diagnosed a somatic delusional disorder. She did not fit the DSM-IV TR diagnostic criteria for anorexia nervosa. Five days after the initiation of oral refeeding and intravenous vitamin K, laboratory testing revealed AST (221 U/L), ALT (539 U/L), alkaline phosphatase (373 U/L), bilirubin (1.3 mg/dL), and INR (1.43). During her initial refeeding period, she had occasional episodes of bradycardia, hypothermia, hypotension, and EKG changes with inverted T waves and prolonged QTc. On hospital day 9, her transaminases were improving but still 5–7 times the upper limit of normal and a liver biopsy was performed. The liver biopsy revealed mild non-specific acute hepatitis with intrasinusoidal and mild centrilobular perivenular fibrosis, focal necroinflammatory foci, acidophil bodies (long arrow), and increased mitotic figures in the hepatic lobules (short arrow) (Figure 1). Dry copper weight was 53.8 ug/g. ATP7B PCR gene testing was negative for Wilson's disease. 

## 3. Discussion

Mild elevations in serum aminotransferase levels have been reported in up to 60% of patients with anorexia nervosa [1]. However, severe acute liver injury with coagulopathy has rarely been described in the Western literature [2–4]. Coagulopathy that is not improved after vitamin K repletion appears to coincide with multiorgan dysfunction as seen in our patient [5–7]. The exact pathogenic mechanism of hepatocyte injury is unclear but has been proposed to be caused by hepatic hypoperfusion or more recently due to hepatocyte autophagy [3, 8, 9]. 

In summary, this is the first reported case of acute liver injury with severe coagulopathy due to protein-calorie malnutrition caused by a somatic delusional disorder. The clinical presentation and liver histology are similar to reported cases of acute liver injury due to anorexia nervosa. Recognition of the patterns of acute liver injury, which can occur in the absence of hypotension in patients with severe protein-calorie malnutrition is important, regardless of the underlying cause. Treatment consists of hemodynamic support, cautious refeeding, and electrolyte replacement. After appropriate refeeding and weight gain of 4 kg, our patient had normalization of transaminases and coagulation factors by hospital day 30.
